# Amyloid Precursor Protein alters Mitochondrial function and Mitophagy

**DOI:** 10.1002/alz.091624

**Published:** 2025-01-03

**Authors:** Taylor A. Strope, Brittany M Hauger, Heather M Wilkins, Kealan Schroeder

**Affiliations:** ^1^ University of Kansas Medical Center, Kansas City, KS USA; ^2^ University of Kansas Alzheimer’s Disease Research Center, Fairway, KS USA

## Abstract

**Background:**

Amyloid Precursor Protein (APP) processing to Aβ is well understood but the function of APP is largely unknown. APP is expressed ubiquitously and localizes to mitochondria. The consequences of mitochondrial APP localization are not known. We leveraged models of altered APP mitochondrial localization to understand the relationship between APP and mitochondrial function.

**Method:**

We have generated several isogenic iPSC lines using Crispr/Cas9 including WT APP (no gene targeting), 3M APP homozygous and hemizygous (which harbors mutations at amino acids +41, +44, and +52 (His to Asp)), APP knockdown (missing one allele), APP knockout (missing both alleles), and APP duplication. These iPSC lines were differentiated into both neurons and astrocytes. Mitochondrial localization of APP was quantified using Western blot analysis. Aβ was measured via ELISA. Mitochondrial respiratory function was analyzed using Seahorse Technology or Vmax spectrophotometric assays. Mitophagy was examined by qPCR against mitochondrial DNA content and an adenovirus expressing EGFP‐COX8. Mitochondrial biogenesis and turnover were measured using an adenoviral MitoTimer vector.

**Result:**

3M APP and knockdown/knockout APP models had reduced mitochondrial APP localization while APP duplication had increased mitochondrial APP localization. Models with reduced APP localization to mitochondria had reduced Aβ production, while APP duplication had increased Aβ production. Reduced mitophagy levels and altered mitochondrial biogenesis were observed in models with reduced mitochondrial APP localization. APP duplication increased mitophagy and altered mitochondrial biogenesis. Both reduced and increased mitochondrial APP localization reduced mitochondrial respiratory function.

**Conclusion:**

APP localization to mitochondria alters mitochondrial function, mitochondrial mass, and mitophagy. Further studies are in progress to elucidate the effects of mitochondrial localization of APP on bioenergetics.

